# The Concept of the Crown and Its Potential Role in the Downfall of Coronavirus

**DOI:** 10.3201/eid2609.AC2609

**Published:** 2020-09

**Authors:** Terence Chorba

**Affiliations:** Centers for Disease Control and Prevention, Atlanta, Georgia, USA

**Keywords:** coronavirus, viruses, severe acute respiratory syndrome coronavirus 2, SARS-CoV-2, coronavirus disease, COVID-19, pandemic, crown, the concept of the crown and its potential role in the downfall of coronavirus, relief showing Helios, sun god in the Greco-Roman mythology, art science connection, emerging infectious diseases, art and medicine, about the cover, respiratory infections, zoonoses

**Figure:**
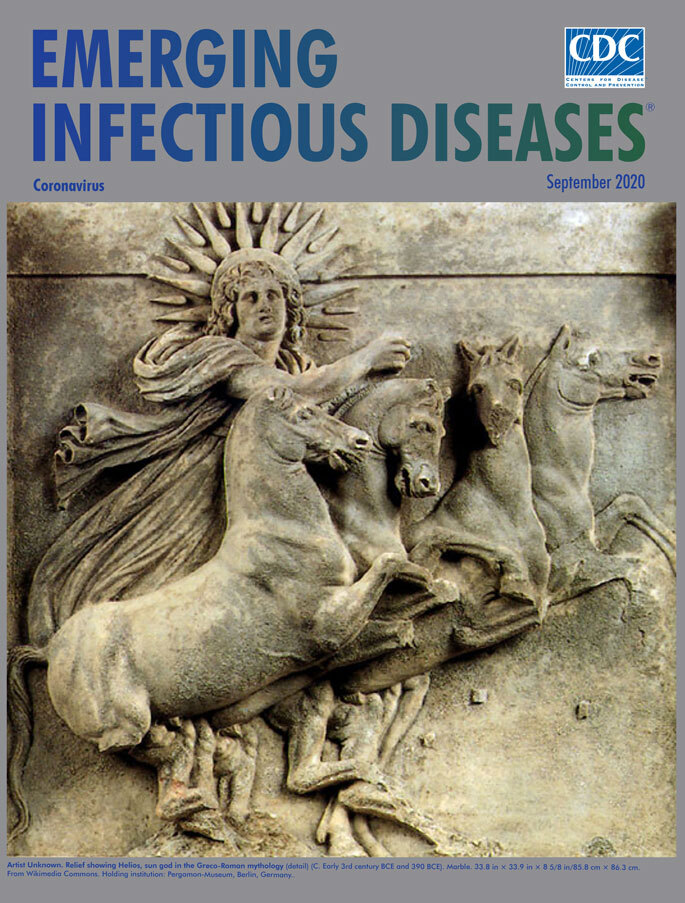
**Artist Unknown. Relief showing Helios, sun god in the Greco-Roman mythology** (c. 390–315 bce). Marble. 33.8 in × 33.9 in × 8 5/8 in/85.8 cm × 86.3 cm. From Wikimedia Commons. Holding institution: Pergamon-Museum, Berlin, Germany

Coronavirus virions are spherical or variable in shape and composed of an outer layer of lipid covered with a crown of club-shaped peplomers or spikes. Within each spike is a helical single-stranded RNA-containing structural protein. Although the term corona was first used in English in the 1500s, it was borrowed directly from the Latin word for “crown.” *Corona* is derived from the Ancient Greek κορώνη *(korōnè),* meaning “garland” or “wreath,” coming from a proto-Indo-European root, ***sker****-* *or* ***ker-*,** meaning “to turn” or “to bend.” 

In the 1967 initial description of an electron microscopic image of a human common cold virus, June Almeida (née Hart) and David Tyrrell described the surface of coronavirus particles as being “covered with a distinct layer of projections roughly 200Ǻ [20 nm] long….[with] a narrow stalk just in the limit of resolution of the microscope and a ‘head’ roughly 100Ǻ across”. In micrographs, the club-shaped spikes that stud the surface of coronaviruses are glycoproteins that give the appearance of a radiate crown. 

**Figure 1:**
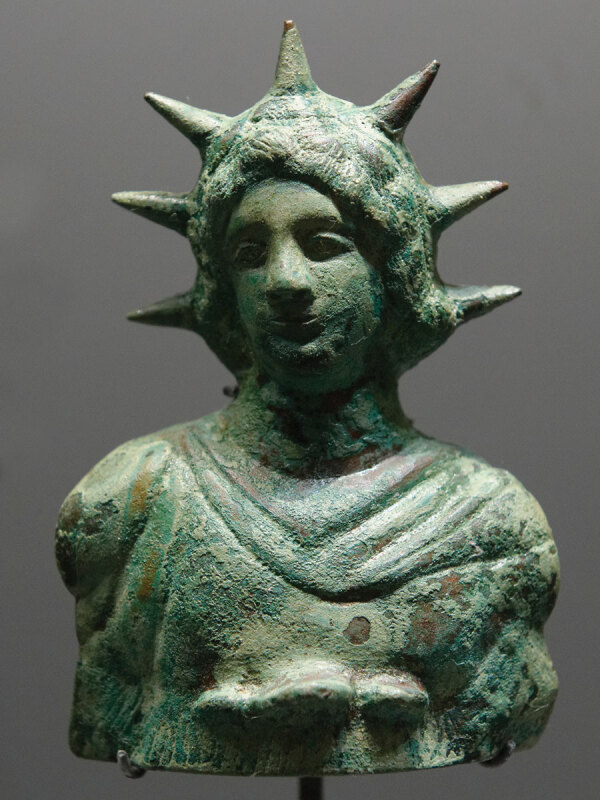
Bust of Helios, radiate (seven rays), with long hair, wearing a chlamys, a short cloak worn by men in ancient Greece. 1st century CE. Public domain image by Marie-Lan Nguyen. Holding institution: Louvre Museum, Paris, France.

Our modern-day *corona* conceptualization of club-shaped spikes on the coronavirus surface comes from traditional representations of crowns as radiate headbands, worn as symbols of sovereign power, to liken that power to that of the sun. Solar deities have been integral in the development of cultures across the world. In predynastic Egypt, Atum was a solar deity associated with the sun god Ra, and Horus was the god of the sky and sun. In Buddhist cosmology, the bodhisattva (one who is on the path toward Buddhahood) of the sun, Sūryaprabha, and the bodhisattva of the moon, *Candraprabha*, are both classically represented as human figures with a background of radiate halos. In traditional Western art, such a solar crown is often represented as a curved band of points representing rays. Representations with radiate crowns date from the 4th century BCE onward, beginning with their frequent inclusion in representations of Alexander III of Macedon (commonly referred to as Alexander the Great), who was likened to the sun deity, Helios (Figure 1). 

Throughout ancient world references, the character of Helios is featured favorably. In the 12th book of the Odyssey, Homer refers to Helios as a god "who gives joy to mortals." This month’s *EID* cover features a rendition of a sculpted metope, a rectangular carved marble plaque in a Doric frieze that was excavated from the Temple of Athena at Troy/Ilion by Heinrich Schliemann in 1872. This metope, dating from the early 4^th^ century BCE, depicts Helios driving a quadriga which is a chariot drawn by four horses abreast. In a later depiction of Helios seen on this page, the deity is represented in a bronze bust with seven rays radiating from a head of long hair. Found at the beginning of the last century in Tripoli, this bust dates from the 1st century CE and may also have been intended to serve as a portrait of Alexander himself. 

**Figure 2:**
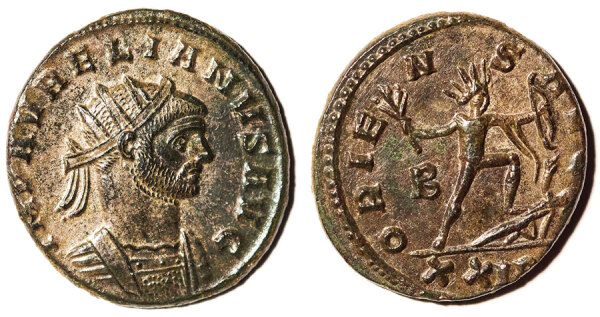
Antoninianus (2 denarii silvered bronze coin) of the Roman emperor Aurelian, 274–275 ce. Obverse: IMP AVRELIANVS AVG [Emperor Aurelian Augustus]. Crowned and cuirassed bust of Aurelian facing right. Reverse: ORIENS AVG [Eastern (rising) sun Augustus]. Crowned figure of *Sol Invictus* [Unconquered Sun] holding laurel branch and bow, stepping on conquered enemy. Private collection, Atlanta, Georgia. Photography by Will Breedlove.

In ancient Rome, the linkage between the power of rulers and the power of the sun was also frequently depicted on coinage. For example, a sovereign wearing a radiate crown on the front (obverse) of the coin and a personification of the sun also wearing a radiate crown and sometimes driving a quadriga on the back (reverse) was common. Figure 2 is an example of such coinage, featuring the emperor Aulerian wearing a radiate crown on the obverse, and, on the reverse, a personification of the official sun god of the later Roman Empire, the *Sol Invictus* (Unconquered Sun), also wearing a radiate crown. In the modern era, positive artistic depictions of liberty and peace have also worn radiate crowns including the Statue of Liberty and the silver US Peace Dollar (1921–1935) that was featured on the March 2018 cover of this journal. 

**Figure 3:**
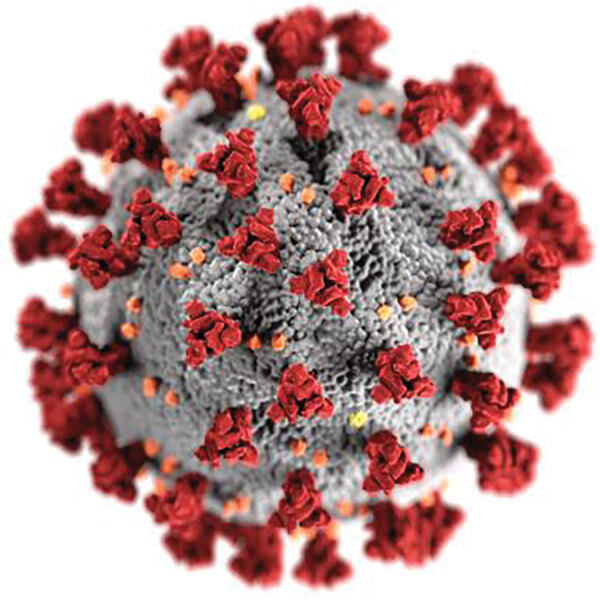
Image from Public Health Image Library, Centers for Disease Control and Prevention, Atlanta, Georgia.

Helios and crowns are associated with power and joy in Western art in ways contradictory to the reality of the tragic pandemic that we are now experiencing with a novel pathogen featuring surface projections that are likened visually to the life-saving rays of the sun. Most recently, 2 illustrators at the Centers for Disease Control and Prevention, Alissa Eckert and Dan Higgins, have immortalized those surface projections in a creative, colored representation of SARS-CoV-2 that itself has “gone viral” in print and digital media (Figure 3). These same spike glycoproteins undergo cleavage into 2 units: a receptor-binding unit (in the globular head of the spike) and a fusion unit (in the stalk of the spike). The receptor-binding unit is responsible for the initial binding of coronaviruses to angiotensin-converting enzyme 2 receptors on the surface of endothelial cells of the human respiratory epithelium and other tissues; the fusion unit mediates the subsequent fusion of the virus with endothelial cell membranes. 

Protruding surface proteins have served as primary targets for successful vaccine development for viruses other than coronavirus, for example the hemagglutinin of influenza A and the surface glycoprotein of Ebola virus. For COVID-19, vaccine trials have begun in which the goal is primarily induction of immunologic responses to the spike protein of the virus *corona*. As is the case with other viruses, interaction of SARS-CoV-2 surface proteins with host cell receptors has also been an important target in the planned development of therapeutic drugs to block viral interactions with host cells. In the face of the current raging COVID-19 pandemic, it is hoped that the spikes of the radiate corona of SARS-CoV-2 will herald its downfall. If these spikes become targets of successful therapeutic and prophylactic interventions, we may somehow resolve the paradox of the resemblance of the spikes of this pathogen to the welcome rays of Helios.
